# GPS Tracking to Monitor the Spatiotemporal Dynamics of Cattle Behavior and Their Relationship with Feces Distribution

**DOI:** 10.3390/ani12182383

**Published:** 2022-09-12

**Authors:** Jessica A. Hassan-Vásquez, Francisco Maroto-Molina, José E. Guerrero-Ginel

**Affiliations:** Department of Animal Production, School of Agricultural and Forestry Engineering (ETSIAM), University of Cordoba, Madrid-Cadiz Rd. km 396, 14071 Cordoba, Spain

**Keywords:** precision livestock farming, extensive farming, agrosilvopastoral systems, environmental impact, ecosystem services

## Abstract

**Simple Summary:**

The environmental impact of livestock production is an important concern of modern societies. In the case of grazing cattle, the accumulation of feces in some areas within paddocks (e.g., around water troughs) may lead to soil degradation. Current precision technologies can monitor grazing animals in (near) real-time to detect and eventually avoid environmental damage. In this paper, we proved that commercial GPS trackers can provide meaningful data on animal distribution and behavior, which can be used to model dung distribution. Model estimates are improved when contextual data (e.g., terrain slope) are considered. The automatic monitoring of dung distribution is an opportunity to improve grazing management and land fertilization, reducing the environmental footprint of cattle production.

**Abstract:**

The sustainability of agrosilvopastoral systems, e.g., *dehesas*, is threatened. It is necessary to deepen the knowledge of grazing and its environmental impact. Precision livestock farming (PLF) technologies pose an opportunity to monitor production practices and their effects, improving decision-making to avoid or reduce environmental damage. The objective of this study was to evaluate the potential of the data provided by commercial GPS collars, together with information about farm characteristics and weather conditions, to characterize the distribution of cattle dung in paddocks, paying special attention to the identification of hotspots with an excessive nutrient load. Seven animals were monitored with smart collars on a *dehesa* farm located in Cordoba, Spain. Dung deposition was recorded weekly in 90 sampling plots (78.5 m^2^) distributed throughout the paddock. Grazing behavior and animal distribution were analyzed in relation to several factors, such as terrain slope, insolation or distance to water. Animal presence in sampling plots, expressed as fix, trajectory segment or time counting, was regressed with dung distribution. Cattle showed a preference for flat terrain and areas close to water, with selection indices of 0.30 and 0.46, respectively. The accumulated animal presence during the experimental period explained between 51.9 and 55.4% of the variance of dung distribution, depending on the indicator used, but other factors, such as distance to water, canopy cover or ambient temperature, also had a significant effect on the spatiotemporal dynamics of dung deposition. Regression models, including GPS data, showed determination coefficients up to 82.8% and were able to detect hotspots of dung deposition. These results are the first step in developing a decision support tool aimed at managing the distribution of dung in pastures and its environmental effects.

## 1. Introduction

Agrosilvopastoral systems combining trees, shrubs, pasture and crops cover large areas on most continents, differing in size and organization between temperate and tropical regions [1]. These production systems have been widely acknowledged for the provision of numerous and diverse ecosystem services, such as food, nutrient cycling, water regulation, biodiversity, etc. [2]. Generally, agrosilvopastoral systems are superior to croplands and grasslands in terms of biodiversity and carbon storage. In fact, estimates of carbon stocks in soil, grass and trees of agrosilvopastoral systems have been used as a basis for the design of climate-smart production systems [3].

*Dehesas*, which occupy more than four million hectares in the Iberian Peninsula, are a classic example of agrosilvopastoral systems. *Dehesas* are derived from the Mediterranean forest ecosystem, consisting of grassland featuring herbaceous species used for grazing cattle and sheep and tree species belonging to the genus *Quercus* (oak), such as the holm oak (*Quercus ilex* L.), although other tree species may also be present. Oaks are protected and pruned to produce acorns, which Iberian pigs feed on during the period called montanera. The understory is usually cleared every seven to ten years to prevent shrub invasion of the grassland. Despite their importance, the economic, ecological and social sustainability of *dehesas* is seriously threatened [4]. *Dehesas* are complex production systems, including both spatial and temporal domains, so their adequate management demands advanced and specific skills. However, the current trend is the reduction of system complexity rather than its management [3], for example, by eliminating crops or shrubs or by reducing pasture diversity, which can severely affect the long-term provision of ecosystem services. Several authors have proposed the continuous monitoring of *dehesas* as a key step to ensure their sustainability [5].

One of the major threats to the sustainability of *dehesas* is the intensification of animal production [4], e.g., by increasing stocking rates or by replacing native breeds with foreign breeds, which are normally less fitted to the seasonal resources of Mediterranean production systems. In addition, the lack of shepherds due to economic and social constraints has led to the generalization of continuous grazing systems, in which cattle graze freely in fenced paddocks for several weeks or months. Under this grazing management strategy, the impact of animal behavior in relation to their preference for certain areas within the paddock is a key issue that has not always been well considered [6,7]. When cattle show strong selective behavior, overgrazing may occur in the preferred spots. In addition, the distribution of animals on pasture has been found to affect soil properties through the accumulation or loss of nutrients both in soils and water [8,9]. The time animals spend in a particular area and their activities in that area may affect the nutrient balance [7,10]. The decomposition of cattle manure is crucial for nutrient cycles altering soil composition, mainly in relation to nitrogen and phosphorus stocks in topsoil [8,11]. Excess nitrogen has been observed in hotspots where animals tend to urinate or defecate more than average [6,12,13]. Other authors have found that microbiota and organic carbon can also be affected by dung distribution [14,15,16,17]. Another important effect of dung deposition on pastures is the reduction of biomass available for livestock feeding, as cattle normally avoid grazing those areas with a high dung density [18]. Additionally, as uneven dung distribution creates heterogeneous nutrient availability in the soil, plant growth and paddock occupation by different plant species can be affected, leading to the accumulation or depletion of undesirable vegetation in different spots [19]. Fertilizers could be reduced in areas with major dung density to homogenize nutrient availability [20]. Disease risk is also associated with feces distribution, as many livestock diseases can be transmitted by oral contact with feces or through fecal aerosols [21].

Scientific literature has identified several factors with a significant effect on grazing behavior and animal distribution, e.g., stocking rate, water and shade location [6], climate conditions or pasture characteristics [7]. Thus, characterizing and understanding animal behavior on pasture is crucial to develop management strategies favoring productivity and, at the same time, avoiding or decreasing the potential negative impact of grazing on the environment [22]. Some authors have pointed out that by predicting or monitoring the sites of livestock dung deposition, the environmental risks associated with an excessive nutrient load can be reduced [23].

Smart technologies applied to animal farming, also known as precision livestock farming (PLF), can provide continuous data to potentially improve farmer decision-making [24,25] in relation to production, reproduction, animal health and welfare and environmental impact [26]. In particular, as visual observation of animals is highly time-consuming and observer-dependent, GPS tracking devices could serve to monitor grazing behavior and animal distribution on pasture, which may help to develop management strategies aimed at reducing an excessive concentration of animals in certain areas, for example, by relocating shade, water or mineral supplementation spots [27].

Several authors have used GPS trackers to characterize cattle behavior, as well as its relationship to dung distribution [15,17,28]. In most cases, the distribution of livestock on pasture was recorded by counting animal visits to certain locations or in landscape units and analyzed by calculating preference indices [29,30,31,32] or home ranges [33]. Few of these studies were carried out in temperate agrosilvopastoral systems, such as *dehesas*. Concerning feces distribution, some authors have correlated the number of GPS positions during a grazing season with the amount of dung in sampling plots of different sizes [2,8]. Most of these studies used high-temporal resolution GPS trackers, which record location fixes every few seconds or minutes. These devices have been proven to be fit for research purposes, but their usefulness under commercial farm conditions is limited due to several reasons, such as robustness, battery life or lack of wireless data transmission [34,35]. However, in recent years, great progress has been made in relation to the development of commercial GPS tracking devices, which are now capable of recording fixes over months or years and transmitting these data through low-power, wide-area (LPWA) networks [36]. The temporal resolution of these commercial devices is typically between 15 min and 2 h.

We hypothesized that, under the conditions of *dehesa* production systems, commercial GPS trackers could be used to monitor the distribution of dung on pasture in near real-time. This information may be useful for implementing management strategies aimed at enhancing nutrient distribution on soils. Thus, the main objective of this study was to evaluate the potential of data provided by commercial GPS devices, together with contextual information about farm characteristics and weather conditions, to describe dung distribution on paddocks, paying special attention to the identification of hotspots with an excessive nutrient load. The optimal spatial and temporal scales for the monitoring of feces distribution were also investigated.

## 2. Materials and Methods

### 2.1. Study Area

The study was carried out on a commercial *dehesa* farm called ‘Las Albaidas’ located in the municipality of Cordoba, Spain. The farm consisted of a single fenced paddock with an area of 23.1 hectares and an average altitude of 150 m above sea level. The coordinates of the paddock centroid were 37.91° N and 4.7° W. The climate can be defined as typical Mediterranean (Csa) according to the Köppen climate classification [37]. The tree canopy cover was 24.7%. The tree layer was mainly composed of oak trees (*Quercus ilex* L.), although some cork oak trees (*Quercus suber* L.) and olive trees (*Olea Europaea* L.) were also present. The shrub layer was almost inexistent, except for paddock borders, which is usual in Spanish *dehesas* dedicated to livestock production. The pasture layer consisted of natural vegetation, including various grass and legume species, e.g., *Dactylis glomerata* L., *Hordeum murinum* L., *Lolium perenne* L., *Poa bulbosa* L., *Medicago arabica* H., *Ornithopus compressus* L. and *Trifolium subterraneum* L. There was a single water trough at the southeastern end of the paddock.

### 2.2. Cattle Monitoring

The farm supported a herd of 18 cattle, aged one to eight years, under a continuous grazing management system. Animals had access to ad libitum mineral supplementation, placed close to the water trough. Breeds were Limousin (males), Retinta and crossbred. Seven animals (two males and five females), which represented 40% of the herd size, were monitored with commercial GPS tracking collars provided by Digitanimal Ltd. (Madrid, Spain) [38]. Animals were selected on the advice of the farmer to be representative of herd behavior. The tracking system included the following components: a GPS unit, a lithium battery pack lasting approximately one year, an IP67 enclosure resistant to water and dust, and an LPWA communications module based on SigFox [39], which enabled the transmission of location fixes from collars to the server in near real-time [36]. GPS devices were configured to gather a location fix of each animal every 30 min if SigFox coverage was available.

### 2.3. Characterization of Dung Distribution

**Figure 1:**
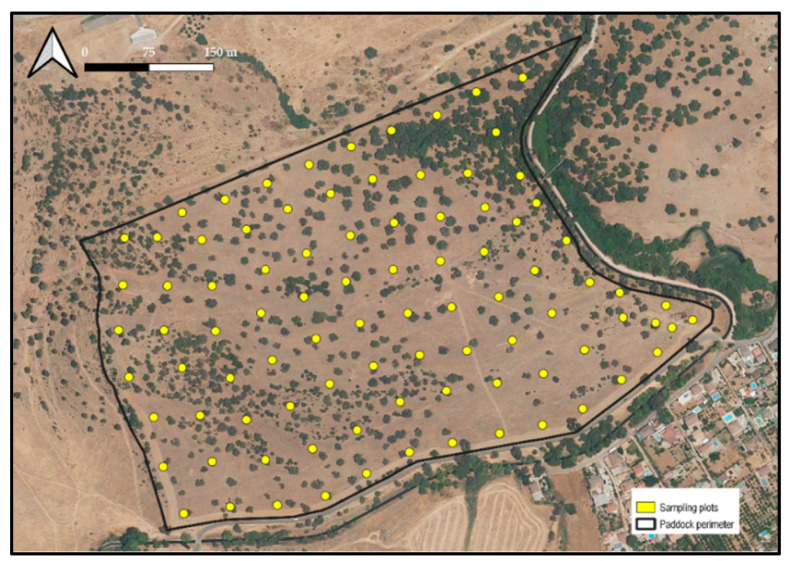
Distribution of dung sampling plots in the study paddock.

Ninety permanent sampling plots were established to measure dung distribution on pasture. Each sampling plot had an area of 78.5 m^2^ (5 m radius) and a separation of approximately 50 m from the other plots. Plot centroids were georeferenced using a Garmin Etrex 10 GPS receiver and marked on the ground with stone piles. Plot distribution in the paddock is shown in Figure 1. The sampled area represented 3.1% of the total area of the paddock.

**Figure 2:**
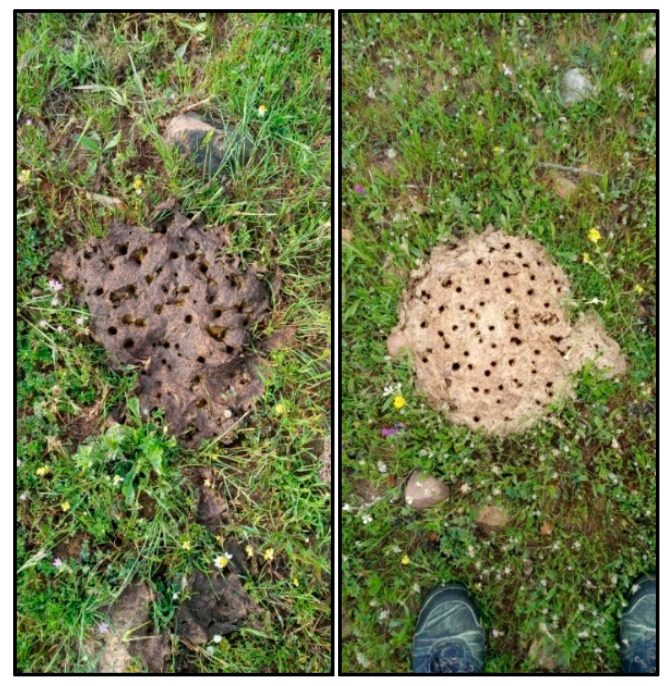
Marking of individual droppings to avoid double counting: fresh dung (**left**) and dry dung (**right**).

The sampling plots were examined weekly from 15 February 2021 (winter) to 2 July 2021 (summer). The number of new feces per week inside each plot was registered in an MS Excel file. Once registered, the feces were marked with a pike to avoid double counting in the following weeks (Figure 2).

### 2.4. Characterization of Factors Affecting the Spatial Variability of Grazing Behavior

Several factors may affect cattle behavior and distribution on pasture, some of which showed temporal variability throughout the study, while others had just spatial variation. Regarding the spatial variability of cattle behavior, the following factors were studied using open data sources: terrain slope, insolation, canopy cover and distance to the single water trough of the paddock.

Slope (%) was calculated for each 5 × 5 m pixel inside the paddock by using the digital model of elevations published by the Spanish National Plan of Aerial Orthophotography (PNOA) [40] and the open-source software QGIS 3.18.1 [41]. Raster pixels were then averaged to match the 10 × 10 m grid of Sentinel 2 images, which were used to characterize vegetation dynamics, as explained in Section 2.5. Insolation, expressed in degrees relative to azimuth, was also calculated for those pixels with a slope greater than 10% using the PNOA digital model of elevations (slopes below 10% were considered flat terrain, with no effect of insolation). A canopy cover shapefile, i.e., the vertical projection of tree crowns on the ground [42], was obtained with QGIS following a semiautomatic procedure consisting of pixel clustering of high-resolution orthophotography (25 × 25 cm), which was also available at the PNOA webpage. A k-means clustering algorithm was used to group orthophotography pixels into 10 categories depending on their values for red, green and blue bands. Afterwards, each cluster was reclassified as “tree” or “grass” based on a visual inspection of the orthophotography, contrasted with field observations. The resulting shapefile was then simplified by eliminating polygons corresponding to the “tree” category with an area below 1 m^2^. This new shapefile was intersected with the Sentinel grid, allowing the calculation of a canopy cover percentage for each pixel. Finally, the Euclidean distance from the centroid of each 10 × 10 m pixel to the water trough of the paddock was computed with QGIS. The resulting maps of slope, insolation, canopy cover and distance to water are included as supplementary materials (Supplementary Materials Figures S1–S4).

Average slope, insolation, canopy cover percentage and distance to the water trough were also calculated for each sampling plot, following the procedure described in the previous paragraph.

### 2.5. Characterization of Factors Affecting the Temporal Variability of Grazing Behavior

Regarding factors influencing the temporal variability of animal behavior, data on weather and vegetation dynamics were collected from open data sources.

**Figure 3:**
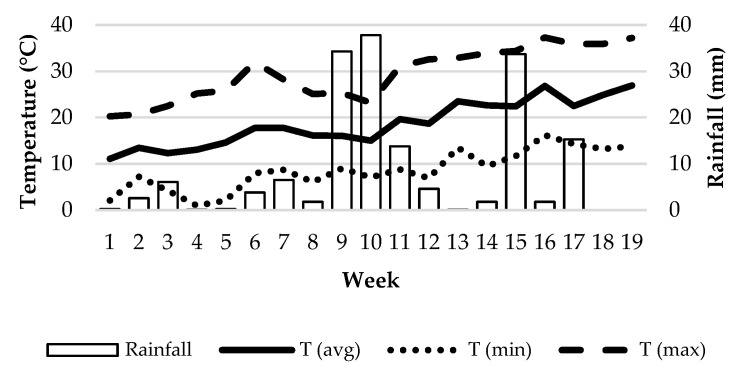
Weekly temperatures and rainfall during the experimental period.

For the characterization of weather conditions during the study, we used the records of an agroclimatic station located at the ‘Rabanales Campus’ [43], which is only 2.1 km from the ‘Las Albaidas’ farm. The following variables were registered daily: average, maximum and minimum temperature (°C), rainfall (mm), average relative humidity (%), total solar radiation (W/m^2^) and average wind speed (m/s). As feces counting was carried out once a week, weather records were aggregated on a weekly basis. Of course, microclimatic differences may have existed within the paddock, which implies the existence of spatial variability for weather data, but these small variations could not be recorded with the available sensors. The main values for temperature and rainfall during the experimental period are shown in Figure 3.

**Figure 4:**
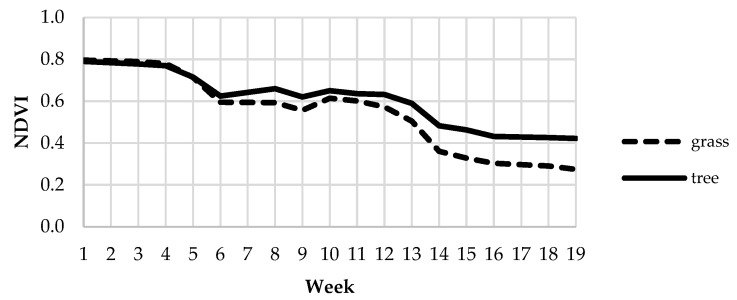
Evolution of NDVI for grass and tree pixels during the experimental period.

On the other hand, we used the spectral data provided by Sentinel 2 satellites [44] for the study of the temporal dynamics of vegetation. Cloud-free images available for the experimental period were downloaded and processed with a custom script implemented in Google Earth Engine [45]. The values of the normalized difference vegetation index (NDVI) for each 10 × 10 m pixel were calculated for the following dates: 15 and 22 February, 12, 14, 17, 22 and 24 March, 1, 3, 16, 18 and 23 April, 1, 6, 11, 13, 16, 18, 26 and 28 May, 2, 7, 10, 12, 20, 25 and 27 June, and 2 July. Weekly NDVI averages were calculated for each pixel. The evolution of the average NDVI in the farm during the experimental period is shown in Figure 4, separately for pasture and tree pixels. As expected, NDVI in tree pixels is higher than in grass pixels at the end of the study, as *Quercus* trees are evergreens.

### 2.6. Data Processing and Analysis

Cattle distribution on the experimental paddock was estimated by counting whole herd location fixes per area unit per week. Area units were landscape polygons, Sentinel grid pixels or sampling plots, depending on the objective of data analysis. Weekly selection indices of the herd were also calculated according to [46]:

$$JSI=\frac{r-p}{r+p-2*r*p}$$

where *JSI* was the value of the Jacobs selection index for a given area unit, *r* was the fraction of location fixes or feces in that area unit, and *p* was the fraction of that area unit in the paddock. Selection indices varied from −1 (negative selection) to +1 (positive selection).

Prior to fix counting, as information provided by commercial GPS collars may contain some missing values due to the lack of SigFox coverage, raw data were preprocessed according to the following steps: (1) fixes located outside the farm border were considered errors and removed; (2) days with one or more devices having three consecutive hours without data were excluded from data analysis; (3) daily trajectories were calculated using the package “trajr” [47] of the open-source software R [48]; and (4) trajectories were resampled to have location fixes at standardized timestamps (every 30 min starting from midnight). After preprocessing, the resulting database had 48 fixes per animal per day, i.e., 336 fixes per animal per week.

Obviously, the temporal resolution of GPS trackers, together with the number of tracking devices, affected the fix counting. To reduce the impact of device configuration on the results of data analysis, we used the number of trajectory segments (straight lines connecting two consecutive location fixes) intersecting each sampling plot as the second indicator of animal presence. Additionally, the length of the intersection between each trajectory segment and each plot was translated into time spent by the animal within that plot by considering that cattle moved at a constant speed between consecutive locations. That is, if the total length of a trajectory segment was “x”, but the intersection of that segment with a particular sampling plot had a length of “y”, the amount of time that the animal spent in that plot was estimated as (30 × y/x) minutes, as each segment referred to 30 min for resampled trajectories.

On the other hand, as cattle performed different activities on pasture, we assigned a distinctive behavior to each GPS fix or trajectory segment. Following the methodology described in previous studies [12,49], the average speed between two consecutive locations was used as an indicator of the type of behavior. On average, those authors found that cattle spend 57.5% of their time resting (including rumination), 40% grazing and 2.5% walking. Thus, percentiles 57.5 and 97.5 for the data distribution of the average speed between consecutive fixes were used as thresholds to classify cattle locations as corresponding to resting (low speed), grazing (medium speed) or walking (high speed).

To model the relationship between cattle and dung distribution, a stepwise regression procedure was used. The number of droppings inside each plot per week was used as the dependent variable, and the number of location fixes per plot per week was used as the independent variable. Alternatively, the number of trajectory segments and the time spent in the plot were also used as independent variables. The effect of separating fixes, segments or times corresponding to resting or grazing behavior was also tested. Data on the slope, insolation, canopy cover, distance to water, meteorological variables and NDVI were included as covariates in all models. A *p*-value below 0.05 was considered statistically significant. To test the effect of spatial and temporal domains on the model, the dependent variable, i.e., the number of droppings per plot, was aggregated spatially (one vs. four plots) and temporarily (one vs. six weeks) in successive rounds of model calibration. Model validation was also performed considering both domains. In the first round, models were calibrated with data corresponding to 70% of the sampling plots and validated with the remaining 30%. Then, data from 70% of the weeks were used for calibration and the last 30% for validation. Statistical calculations were performed in IBM SPSS Statistics 25 (Armonk, NY, USA) and R.

## 3. Results

### 3.1. Effects of Spatial Patterns on Cattle and Dung Distribution

A total of 1838 fixes were located inside the 90 sampling plots during the experimental period, meaning 4.1% of all fixes were gathered by GPS collars. These data were considered representative of cattle behavior within the whole paddock (as explained in the Material and Methods section, sampling plots represented 3.1% of the paddock area).

However, as shown in Table 1, the distribution of animals in the paddock was not even. Although only 35.7% of the available land could be considered flat (slope below 10%), 50.8% of location fixes were found in flat areas. Disproportionality was even larger for feces distribution, as 62.5% of droppings were deposited in these areas. This was reflected in the weekly values of the Jacobs selection index, which averaged 0.30 and 0.45 for location fixes and droppings, respectively. Regarding insolation, slopes facing south were especially avoided by cows, both for location and defecation. On the other hand, a significant reduction in the number of dung pats was observed in slopes facing west compared to the number of fixes. According to GPS timestamps, cattle used those slopes mostly for evening grazing during late spring and summer, while most droppings were concentrated in resting areas. Tree layer did not affect animal distribution, as pixels with a high canopy cover showed a similar selection index to those pixels without trees (a significantly higher index was found for areas with a canopy cover between 25 and 50%, but all values of selection indices were very close to zero, indicating no animal preference). However, canopy cover did affect dung distribution, as the selection index for droppings in areas without trees was positive and significantly different from covered areas. Finally, a strong preference for areas closer than 100 m to the water trough was observed. Most location fixes in these areas corresponded to resting behavior during the morning, part of which could correspond to rumination. For night rest, cattle preferred flat and elevated areas located between 350 and 550 m from the water trough. The effect of distance to water on the distribution of dung was significantly higher than on animal distribution, as 42.6% of droppings (19.76% of cattle positions) were registered in sampling plots closer than 100 m to the trough, while this area represented only 8.7% of the available land.

### 3.2. Effects of Temporal Patterns on Cattle and Dung Distribution

**Figure 5:**
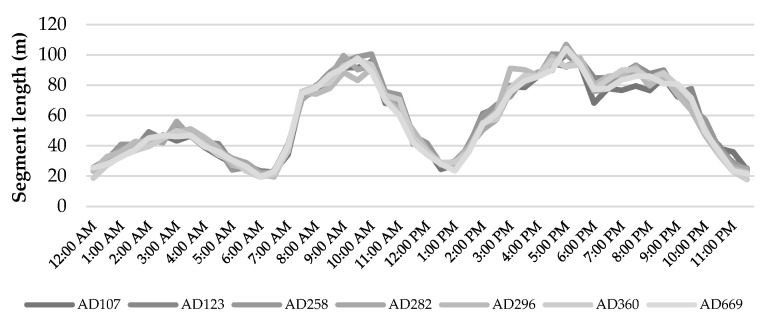
Individual variability of the daily activities of cattle.

Animal behavior and distribution on pasture showed a strong temporal component. Figure 5 shows the average length of trajectory segments within a day for each monitored animal. Segments below 50 m, which corresponded to an average speed of 1.6 m/min, were associated with resting behavior. Two main resting bouts around midday and during the night were observed in all animals. Accordingly, two main grazing bouts were also identified, one in the morning and one in the afternoon/evening. Differences among animals were not significant.

**Figure 6:**
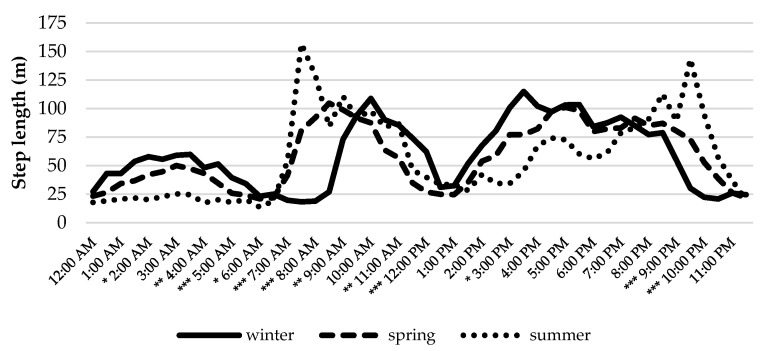
Seasonal variability of the daily activities of cattle. Asterisks indicate statistical significance: * = *p* < 0.05; ** = *p* < 0.01; *** = *p* < 0.001.

Daily activity patterns were not constant during the experimental period. Significant differences in the average length of trajectory segments at various timestamps were observed among seasons, as shown in Figure 6. The morning grazing period was longer and occurred earlier during summer, while the second grazing period was shorter and delayed until 9–10 p.m. In contrast, animals showed less activity at sunrise and longer grazing periods in the afternoon during winter. This adaptative behavior possibly responded to higher temperatures in summer and daylight, which increased approximately 3 h between the beginning and end of the experimental period.

**Figure 7:**
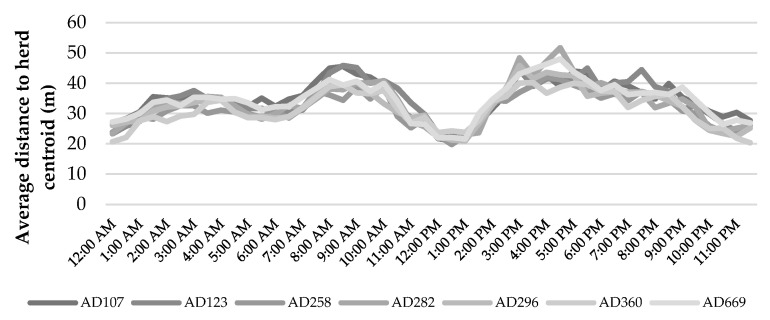
Individual variability of cattle behavior in relation to the herd.

**Figure 8:**
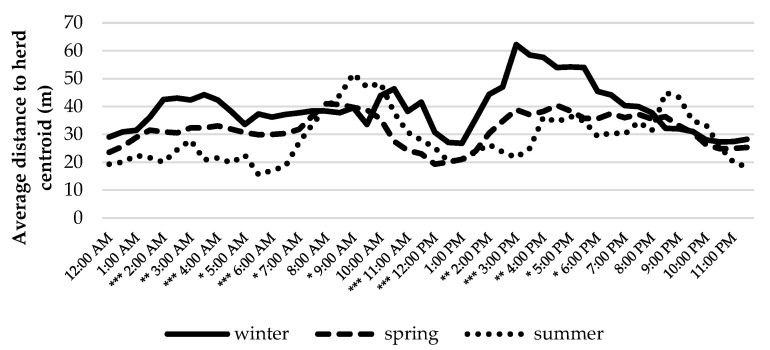
Seasonal variability of herd behavior. Asterisks indicate statistical significance: * = *p* < 0.05; ** = *p* < 0.01; *** = *p* < 0.001.

Herd dispersal while performing different activities, which was estimated as the average distance between individuals and herd centroid, is another indicator of animal distribution. It is worth noting that the estimation of herd dispersal requires that the location fixes from all animals are synchronized, which is not the case with commercial devices. Then, as it was done in this study, the calculation and resampling of trajectories at standardized timestamps are needed. Figure 7 and Figure 8 show the intraday dynamics of the distance to herd centroid by animal and by season. The herd was more dispersed for grazing than for resting, and no significant differences among individuals were observed. Curiously, distance to herd centroid was significantly lower during diurnal resting (partly rumination) than for night rest. With regard to the effect of the season, the herd was significantly more dispersed during winter, especially for the afternoon/evening grazing bout. This effect could respond to the type of vegetation available during this season, which encouraged cattle to seek high-quality pastures.

**Figure 9:**
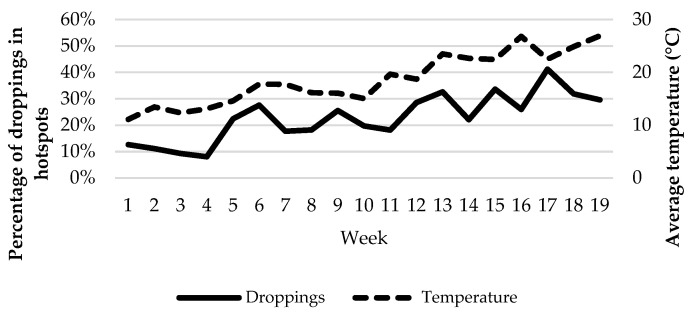
Weekly percentage of droppings in the two main hotspots for dung deposition according to ambient temperature.

**Figure 10:**
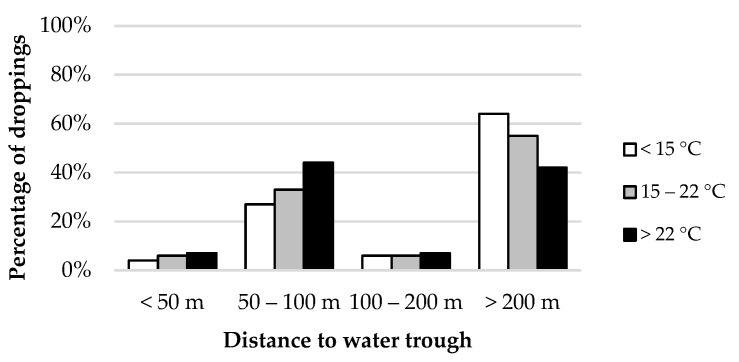
Effect of the interaction ambient temperature-distance to water in dung distribution.

The temporal variability of animal distribution influenced the distribution of dung. Two single sampling plots located in a flat area between 50 and 100 m from the water trough, which represented 2.2% of the total area sampled, accumulated 24.3% of droppings during the whole study period (from now on, we refer to these two plots as hotspots). However, that percentage differed among weeks, ranging from 8.0% to 41.2% (Figure 9). Such percentage was increased with ambient temperature due to the higher presence of animals in areas close to the water during warm days (Figure 10). While animals spent most of their time in areas far from the water trough (>200 m) during days with an average temperature below 15 °C, time was equally distributed between far areas and those situated between 50 and 100 m from water when the average temperature rose to 22 °C and above.

**Figure 11:**
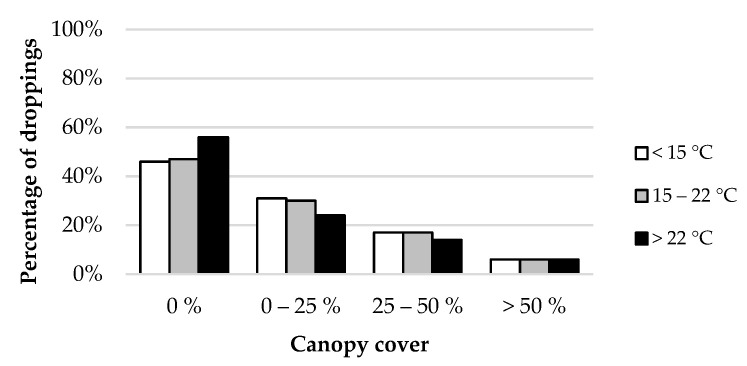
Effect of ambient temperature-canopy cover interaction in the distribution of dung.

An interaction between ambient temperature and canopy cover was also observed for the distribution of dung (Figure 11). Although most droppings were deposited in areas without tree cover, this proportion was even larger on warm days. Cattle may spend more time under trees with higher temperatures, but it did not increase dung deposition in these areas. In contrast, the number of dung pats was reduced in plots with some canopy cover.

### 3.3. Relationships between GPS Data and Dung Accumulation in Sampling Plots

**Figure 12:**
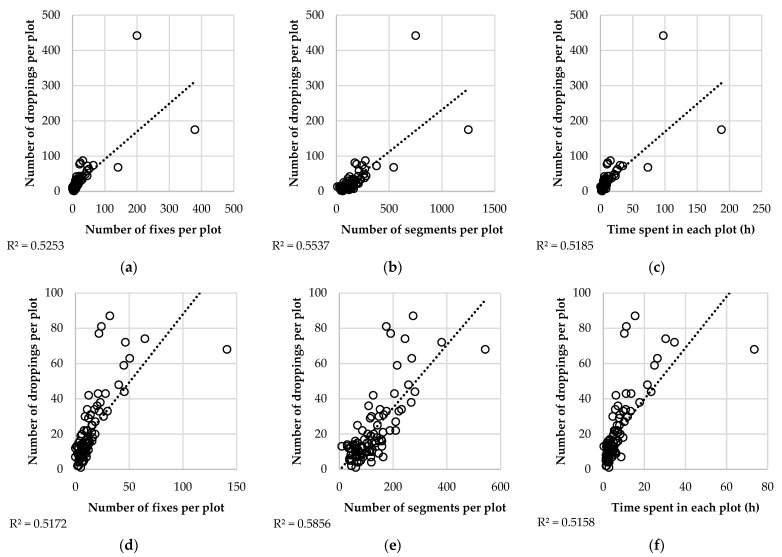
Relationships between feces counting and animal presence estimated as location fix counting (**a**,**d**), trajectory segment counting (**b**,**e**) or time spent inside plot (**c**,**f**), including (**a**–**c**) and excluding (**d**–**f**) hotspots.

Figure 12 shows the relationship between the number of feces accumulated in each sampling plot during the experimental period and animal presence in those plots. The presence of animals was represented with three different indicators: number of location fixes, number of trajectory segments, and total time animals spent within the plot. As hotspots accumulated a large proportion of feces and location fixes, they could have a great leverage effect on the regression between feces counting and animal presence. To check that, Figure 12 includes the relationship between feces and animal presence calculated both including (Figure 12a–c) and excluding (Figure 12d–f) hotspot data.

The coefficients of determination were similar in all cases, ranging from 0.5 to 0.6. Nonetheless, slightly better results were obtained when the number of segments intersecting each plot was used as the independent variable. In Figure 12a–c, the hotspots accumulating most of the feces can be easily identified. The total count of dung pats in hotspots was 175 and 442 for the whole period, i.e., 2.2 and 5.6 droppings per m^2^ for 19 weeks. As hotspot data had a large leverage effect on the relationship between feces counting and animal presence, Figure 12d–f shows the correlation between both variables, excluding hotspots. The values of coefficients of determination were not greatly affected when hotspot data were excluded, although the relationship between the number of droppings and the number of segments was, in fact, improved and showed the highest linearity.

**Figure 13:**
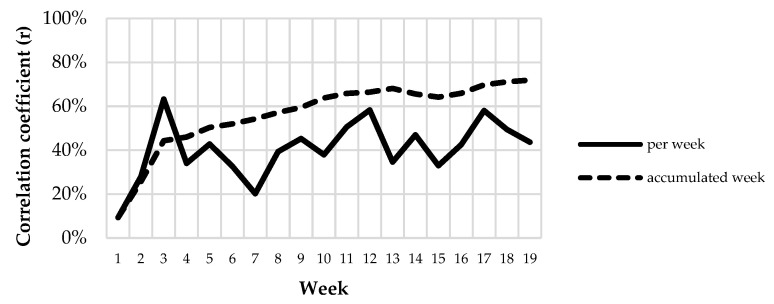
Correlation coefficient between the number of droppings and the number of location fixes per plot, per week and per accumulated week.

**Figure 14:**
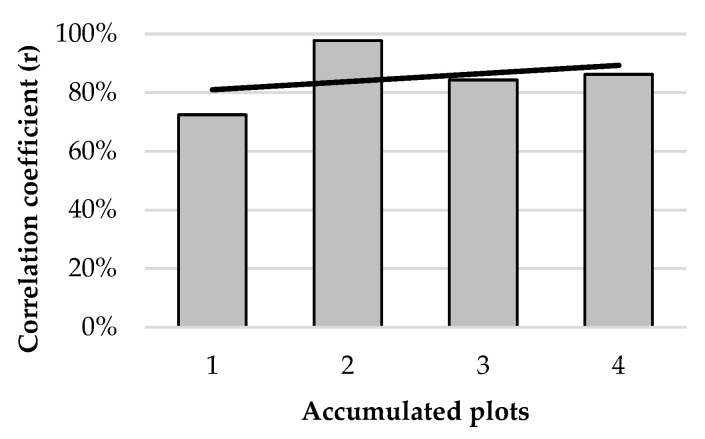
Correlation coefficient between the number of droppings and the number of location fixes per accumulated plot.

Figure 13 and Figure 14 analyze the temporal and spatial domains of the relationship between dung distribution and animal presence. The correlation coefficient between the number of droppings and the number of location fixes per plot per week showed a large variation among weeks, ranging from 0.09 in week 1 to 0.63 in week 3. When using accumulated weeks, the maximum correlation coefficient (0.72) corresponded to the sum of data from the 19 weeks of the study, but r > 0.6 was reached with just 10 weeks and r > 0.5 with 6 weeks. Regarding the spatial component of the correlation between the studied variables, when data were aggregated from 1 to 4 plots (close to each other), the correlation coefficient values were only slightly improved, meaning that the temporal domain was of greater importance for models correlating the distributions of dung and animals.

### 3.4. Calibration and Validation of Prediction Models

To evaluate the possibilities of tracking the distribution of dung in near-real time based on data from commercial GPS collars and open data sources, various models were tested.

**Figure 15:**
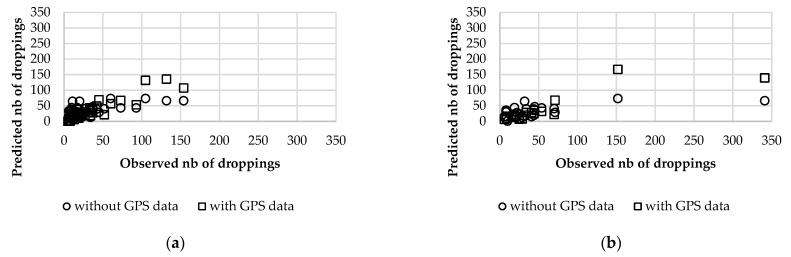
Relationship between observed and predicted number of droppings for the best model in Table 2 and for the equivalent model excluding GPS data. (**a**) Calibration data set. (**b**) Validation data set.

Table 2 includes the constant, coefficients and performance statistics of prediction models calibrated with data from weeks 1 to 13 and validated with data from weeks 14 to 19. The number of location fixes, the number of trajectory segments and the time spent in sampling plots were alternatively used as input variables. Models obtained without considering data from GPS collars were also calculated. To evaluate the effect of spatial and temporal domains in performance, models were calculated with data per plot and per week and with data aggregated for 4 plots and 6 weeks, considering all the possible combinations. Model performance changed little when considering fixes, segments or time as independent variables, although errors were consistently lower for models using trajectory segments. In some cases, the stepwise procedure selected the number of fixes or segments corresponding to resting behavior as more significant to predict the number of droppings than the total number of fixes/segments, suggesting that dung deposition may preferably occur in resting areas. Covariates included in prediction models differed between rounds, although distance to water and slope were included in most models. It is worth noting that the sign of the coefficients of these contextual variables was always negative, which means that the relationship between dung distribution and the presence of animals was lowered by increasing slope and distance to water. Canopy cover was significant only when data were aggregated for 4 plots. Errors were significantly reduced by including data from GPS collars in prediction models, showing their added value for tracking dung distribution, and by aggregating data both in the spatial and temporal domains. The best model was obtained with data aggregated for 4 plots and 6 weeks and with trajectory segments as the independent variable. Figure 15 shows the relationship between the observed and predicted number of droppings using this model, both for calibration and validation data sets. In general, validation errors were significantly higher than those corresponding to calibration, showing that the relationship between feces counting and independent variables was different during the first and the last weeks of the trial. In fact, as explained in previous sections, the ambient temperature increased at the end of the experiment, altering the effects of distance to water and canopy cover in the relationship between dung distribution and animal presence. Figure 15 shows that the best model was not able to adequately predict the number of feces in the two hotspots (each one including 4 sampling plots), which accumulated more than 300 droppings in the last 6 weeks of the experiment. In fact, the maximum number of droppings in the calibration dataset was 154. However, the inclusion of GPS data in the model did allow the identification of the two hotspots, as they were the only plots with more than 100 predicted droppings. The model including uniquely contextual variables did not differentiate hotspots from other spots of the paddock. However, the generalization of prediction models for dung distribution would require data from additional weeks in order to characterize all possible interactions between independent variables. Obviously, data from other farms, stocking rates, grazing management methods, etc., would also be needed.

Table 3 shows the results for models calibrated with 70% of the plots and validated with the remaining 30%. In this case, the selected variables were less consistent among models, showing that 70% of the plots may be insufficient to characterize the spatial component of the relationship between dung distribution and the presence of animals. Furthermore, errors were higher than for models in Table 2.

## 4. Discussion

In recent years, several authors have pointed out the need to deepen the knowledge of cattle grazing behavior and its environmental impact [13,16,50], both in terms of differentiating individual and herd behavior and improving grazing management strategies [51]. This study proposes several approaches to understanding and quantifying grazing patterns using PLF technologies. Unlike previous studies [34,35,52], research was conducted by combining commercial GPS devices and open data sources, which makes results easily implementable in real-world livestock farming. Although the data provided by commercial devices presented some limitations, they could be overcome with specific data processing strategies, which allowed the extraction of meaningful information on animal behavior and distribution in pastures.

Open data sources, such as digital models of elevations, orthophotographs and satellite images, were especially useful for identifying and characterizing the sites preferred by animals to perform different activities. In the present study, cattle showed a strong preference for flat terrain and areas near water, in agreement with previous studies [27,53,54]. Consequently, these factors influenced the distribution of dung within the paddock. [55,56] concluded that, in warm environments, the location of water and shade influences the behavior and distribution of animals. However, in our study, the availability of shade under trees, measured as canopy cover, did not affect the distribution of animals, although it did influence the dispersal of dung. However, contrary to what was observed by [2,57], in this study, fewer cattle droppings were registered in pixels with high canopy cover.

The temporal domain was also important to describe the distribution of animals and dung. Large variability existed both intraday and between days. In this study, the interactions among ambient temperature, distance to water and canopy cover explained the dynamics of animal distribution and their relationship with feces deposition. Contrary to what was observed in previous studies [27,53], relative humidity did not significantly influence dung dispersal in our trial, probably because of its limited range when aggregated weekly.

Regarding herd behavior, our results coincide with [58,59], who showed that the distance of individuals to herd centroid was affected by the time of the year. We also observed that this distance depended on the type of activity carried out by animals, with a maximum average distance during grazing.

As the spatial and temporal patterns of dung distribution are not uniform [2,18,60,61], several studies [20,21] have pointed out the need to develop tools aimed at the implementation of management strategies able to modify animal distribution, decreasing overgrazing and nutrient accumulation in small areas. The strategic location of water, shade, salt or mineral supplementation points could help to modify animal distribution [27,62]. According to [8], the prediction of sites of dung accumulation could be used to reduce the risk of contamination of water and soil. Fertilizer use could be reduced on gentle slopes, areas around fences and salt ponds, etc., avoiding the negative effects of excess nutrients [20].

There are not many previous studies on modelling the relationship between animal presence and dung deposition [53,54], and none of them were done under Mediterranean conditions. However, our results are in accordance with the findings of [53], who identified the distance to water as a significant factor to be included in models, especially in warm seasons. Various authors [28,63] also identified climate conditions and topography as key factors. Furthermore, our models were able to identify hotspots of dung deposition, which was pointed out as very important by [2,57,60]. Identification of hotspots was only possible when GPS data were included in models, which demonstrates the value of this information in improving decision-making in relation to pasture management. The temporal and spatial scales of the data used to build models, which were not addressed by most of the previous studies, were found to be an important factor to consider. In this study, the best results were obtained from data aggregated in 4 sampling plots (314.2 m^2^) and 6 weeks, which are adequate scales for decision-making in relation to grazing management and pasture fertilization in Mediterranean environments.

In this study, regression models have been used to describe the relationship between cattle presence characterized through GPS collars and dung distribution. Previous studies on this topic have also used regression analysis [2,20,61], although more complex procedures, such as generalized additive models or machine learning algorithms, would be expected to better predict the distribution of dung. However, since the goal of this study was not to develop final models but to understand the importance of integrating several data sources in predicting the distribution of dung, regression models were preferred because of their interpretability.

In summary, the findings of the present study are the first step in developing decision-making tools aimed at mitigating the environmental impact of livestock production, which has been a demand of farmers and other stakeholders for a long time [63].

## 5. Conclusions

The results obtained in the present study confirmed, in a Mediterranean environment, the importance of several factors in explaining the behavioral patterns of grazing cattle. Precision livestock farming (PLF) tools have proven to be useful for characterizing grazing patterns in *dehesa* ecosystems, which can be extrapolated to other agrosilvopastoral systems. The integration of open data, such as meteorological records, topographic models and satellite imagery, which are available at different scales for most grazing territories around the world, proved to be useful for the study of the effect of several factors on animal distribution, as well as on its relationship with feces spreading.

Data processing was important to extract value from the information provided by commercial devices and open data sources. The consideration of the temporal and spatial scales also had a large impact on the results of the study. Further research is needed to include diverse production systems and management strategies in model calibration data sets, which could make models more general and applicable under different conditions.

The relationship between animal presence measured by GPS collars and dung distribution was not straightforward, as the activities performed by animals at each site, as well as site characteristics, also affected dung deposition. However, the inclusion of GPS data in regression models allowed the identification of hotspots of dung accumulation, which was not possible using only contextual information.

## Figures and Tables

**Table 1:** Animal and dung distribution within the experimental paddock according to several factors.

Factor	Category	% Total Area	% Total Fixes	% Total Droppings	JSI Fixes	JSI Droppings	Sig.
Slope	<5%	7.42	13.49	20.05	0.29 ± 0.15 ^c^	0.36 ± 0.19 ^c^	
	5–10%	28.23	37.37	42.46	0.20 ± 0.08 ^c^	0.29 ± 0.14 ^c^	*
	10–20%	53.29	44.72	34.69	−0.17 ± 0.12 ^b^	−0.36 ± 0.14 ^b^	***
	>20%	11.06	4.41	2.80	−0.48 ± 0.18 ^a^	−0.54 ± 0.28 ^a^	
Insolation	Flat	35.65	50.86	62.51	0.30 ± 0.13 ^c^	0.45 ± 0.13 ^c^	***
	North	12.82	8.06	11.17	−0.27 ± 0.15 ^b^	−0.18 ± 0.18 ^b^	
	East	9.12	6.37	7.06	−0.21 ± 0.16 ^b^	−0.26 ± 0.20 ^b^	
	South	2.74	0.98	2.25	−0.51 ± 0.22 ^a^	−0.49 ± 0.28 ^a^	
	West	39.66	33.72	17.01	−0.13 ± 0.16 ^b^	−0.37 ± 0.10 ^ab^	***
Canopy cover	0%	26.89	25.59	51.30	−0.03 ± 0.08 ^a^	0.21 ± 0.15 ^b^	***
	0–25%	33.74	34.23	27.35	0.01 ± 0.05 ^ab^	−0.15 ± 0.14 ^a^	***
	25–50%	19.65	20.97	15.31	0.04 ± 0.05 ^b^	−0.09 ± 0.21 ^a^	*
	>50%	19.73	19.22	6.04	−0.02 ± 0.10 ^a^	−0.24 ± 0.24 ^a^	**
Distance to water	<50 m	2.22	3.55	5.92	0.20 ± 0.18 ^c^	0.20 ± 0.29 ^b^	
	50–100 m	6.45	16.21	36.70	0.46 ± 0.11 ^d^	0.64 ± 0.12 ^c^	***
	100–200 m	15.62	8.81	6.43	−0.32 ± 0.07 ^a^	−0.50 ± 0.20 ^a^	***
	>200 m	75.71	71.43	50.95	−0.10 ± 0.11 ^b^	−0.36 ± 0.18 ^a^	***

Abbreviations: JSI = Jacobs selection index (calculated per week). Different superscript letters within the same column indicate statistical significance (*p* < 0.05). Asterisks indicate statistical significance within the same row: * = *p* < 0.05; ** = *p* < 0.01; *** = *p* < 0.001.

**Table 2:** Prediction models validated in the temporal domain.

Spatial Domain	Temporal Domain	GPS Variable	CONS	Coefficients *						R^2^	MAEc **	MAEv **
				GPS	CC	DW	IN	NDVI	SL			
1 plot	1 week	-	3.382			−0.003			−0.097	0.097	1.324	1.912
1 plot	1 week	fix	2.172	0.479 ***		−0.002			−0.059	0.321	1.116	1.646
1 plot	1 week	segment	1.872	0.186 ***		−0.002			−0.049	0.356	1.068	1.625
1 plot	1 week	time	1.896	0.935		−0.002			−0.053	0.320	1.092	1.625
1 plot	6 weeks	-	21.895			−0.020			−0.633	0.156	1.002	1.503
1 plot	6 weeks	fix	11.282	0.512		−0.009			−0.322	0.508	0.709	1.116
1 plot	6 weeks	segment	−0.576	0.173						0.539	0.687	1.113
1 plot	6 weeks	time	4.879	1.199	−6.785					0.512	0.862	1.202
4 plots	1 week	-	18.670			−0.013	−0.012		−0.586	0.291	0.864	1.400
4 plots	1 week	fix	5.846	0.697 ***	−12.760	−0.003		20.025		0.529	0.679	1.146
4 plots	1 week	segment	4.930	0.250 ***	−10.092	−0.003		14.484		0.576	0.636	1.085
4 plots	1 week	time	3.595	1.363	−10.286			16.476		0.526	0.665	1.104
4 plots	6 weeks	-	99.713			−0.087			−3.336	0.380	0.737	1.303
4 plots	6 weeks	fix	20.231	0.757	−56.944					0.743	0.440	0.879
4 plots	6 weeks	segment	19.528	0.299 ***	−46.765					0.828	0.363	0.802
4 plots	6 weeks	time	17.450	1.590	−48.280					0.773	0.399	0.850

* Abbreviations: GPS = number of fixes, segments or time sum, CC = canopy cover, DW = distance to water, IN = insolation, NDVI = normalized difference vegetation index, SL = slope. ** Mean absolute error (calibration and validation) expressed per plot and week. *** Data referred only to resting behavior.

**Table 3:** Prediction models validated in the spatial domain.

Spatial Domain	Temporal Domain	GPS Variable	CONS	Coefficients *								R^2^	MAEc **	MAEv **
				GPS	CC	DW	IN	NDVI	SL	TA	TM			
1 plot	1 week	-	2.016		0.014	−0.003			−0.103	0.064		0.167	1.207	2.128
1 plot	1 week	point	1.742	0.366	−0.005	−0.002	−0.002		−0.061	0.052		0.451	1.058	1.984
1 plot	1 week	segment	1.033	0.122		−0.001	−0.003		−0.055	0.045		0.470	2.237	3.003
1 plot	1 week	time	1.769	0.797	−0.007	−0.001	−0.003		−0.058	0.049		0.471	1.100	2.063
1 plot	6 weeks	-	−9.061		9.141	−0.021			−0.651		0.865	0.293	0.934	1.790
1 plot	6 weeks	point	−8.697	0.408	−4.843	−0.009	−0.017		−0.356		0.747	0.746	0.587	1.558
1 plot	6 weeks	segment	2.686	0.142			−0.024	−18.290	−0.302	0.394		0.779	0.527	1.635
1 plot	6 weeks	time	−7.385	0.839	−4.902	−0.009	−0.018		−0.354		0.710	0.760	0.554	1.538
4 plots	1 week	-	29.546		−21.261	−0.016	−0.058	−30.183	−0.715	0.333		0.360	1.241	1.603
4 plots	1 week	point	11.772	0.913 ***	−29.115		−0.036	−19.093		0.217		0.590	0.900	1.234
4 plots	1 week	segment	9.053	0.332 ***	−22.751		−0.029	−18.016		0.197		0.641	0.844	1.124
4 plots	1 week	time	11.994	1.850 ***	−28.989		−0.036	−17.675		0.197		0.600	0.894	1.223
4 plots	6 weeks	-	129.717			−0.167			−3.777			0.323	1.170	0.886
4 plots	6 weeks	point	31.117	1.073 ***	−93.624							0.657	0.722	0.572
4 plots	6 weeks	segment	12.547	0.393 ***								0.707	0.597	0.557
4 plots	6 weeks	time	31.002	2.140 ***	−92.531							0.667	0.712	0.522

* Abbreviations: GPS = number of fixes, segments or time sum, CC = canopy cover, DW = distance to water, IN = insolation, NDVI = normalized difference vegetation index, SL = slope, TA = average temperature, TM = maximum temperature. ** Mean absolute error (calibration and validation) expressed per plot and week. *** Data referred only to resting behavior.

## Data Availability

The data presented in this study are available on request from the corresponding author. The data are not publicly available because they mostly refer to animal coordinates in a commercial farm.

## Supplementary Materials

The following supporting information can be downloaded at: https://www.mdpi.com/article/10.3390/ani12182383/s1, Figure S1: Slope values per pixel in the paddock; Figure S2: Insolation values per pixel in the paddock; Figure S3: Canopy cover values per pixel in the paddock; Figure S4: Distance to water values per pixel in the paddock.
