# Supplementary material for: GPS Tracking to Monitor the Spatiotemporal Dynamics of Cattle Behavior and Their Relationship with Feces Distribution
Source: Animals (Basel). 2022 Sep 12;12(18):2383. doi: 10.3390/ani12182383 (PMC9495034; doi:10.3390/ani12182383)
Supplement: Supplementary file 1 [file animals-12-02383-s001.zip › animals-1830853-supplementary.pdf]

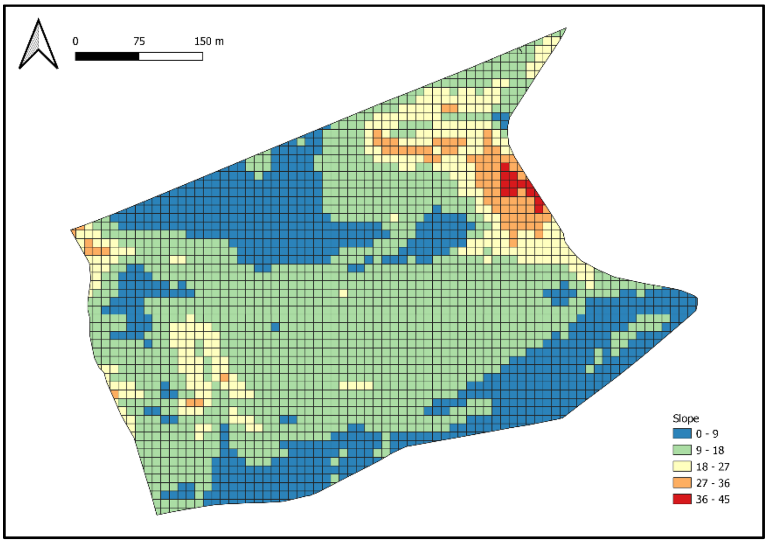

Figure S1: Slope values per pixel in the paddock.

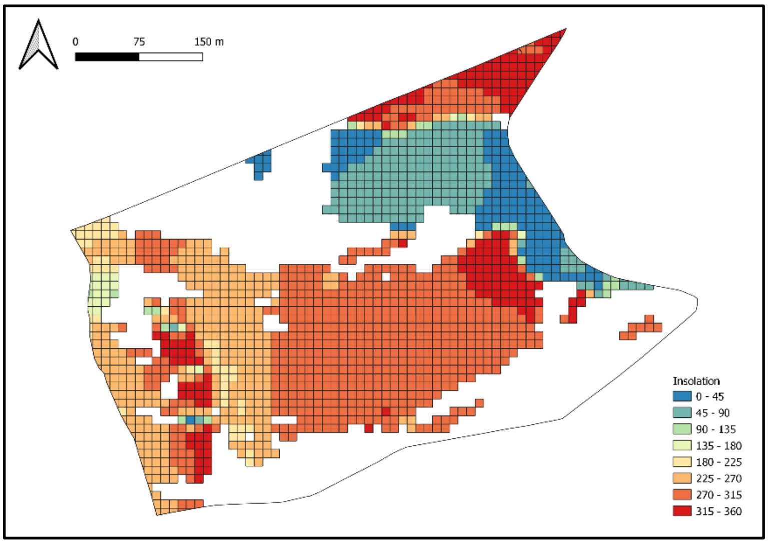

Figure S2: Insolation values per pixel in the paddock.

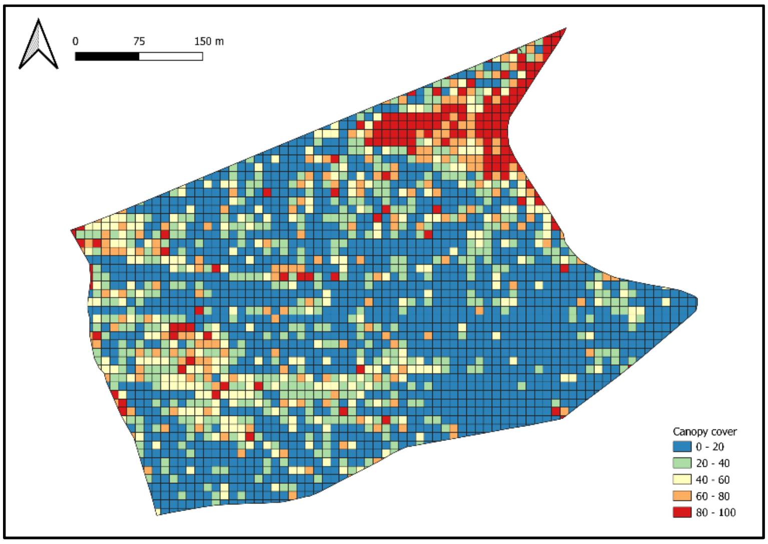

Figure S3: Canopy cover values per pixel in the paddock.

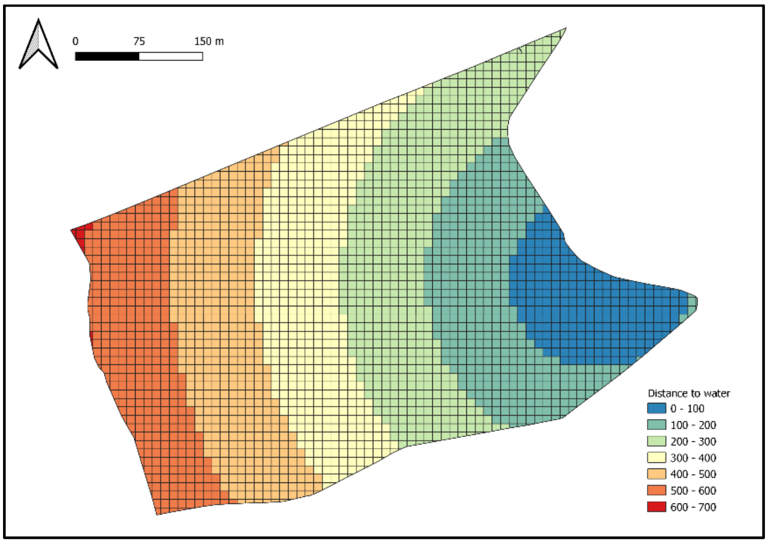

Figure S4: Distance to water values per pixel in the paddock.
